# A comparative study of state self-esteem responses to social media feedback loops in adolescents and adults

**DOI:** 10.3389/fpsyg.2025.1625771

**Published:** 2025-09-23

**Authors:** Yu-Hsing Chen

**Affiliations:** School of Journalism and Communication, Jinan University, Guangzhou, China

**Keywords:** digital identity regulation, self-concept development, feedback processing, emotional reactivity, authenticity in self-presentation, adolescent vulnerability

## Abstract

**Introduction:**

Social media platforms provide constant, quantifiable feedback that can shape self-esteem, particularly during adolescence, a period of heightened neurobiological sensitivity to social evaluation. While previous research has examined digital feedback effects on well-being, comparative evidence on adolescents and adults remains limited. This study investigated how feedback valence, social comparison, and perceived authenticity influence state self-esteem across these developmental groups.

**Methods:**

A cross-sectional, quasi-experimental design was employed with 240 urban Chinese participants (120 adolescents aged 13–18 years and 120 adults aged 25–40 years). Participants were randomly assigned to positive, neutral, or negative feedback conditions within a simulated social media environment. State self-esteem was assessed using the State Self-Esteem Scale, with social comparison orientation and perceived authenticity measured as potential mediating and moderating factors. Data were analyzed using ANOVA, mediation, and moderation models with covariate controls.

**Results:**

Adolescents demonstrated significantly greater sensitivity to feedback than adults, with larger increases in self-esteem after positive feedback and sharper decreases after negative feedback (Age × Valence interaction, *F*(2,234) = 6.65, p = 0.002). Main effects of feedback valence were observed across both groups (*F*(2,237) = 10.85, p < 0.001). Mediation analyses indicated that social comparison orientation partially accounted for the relationship between feedback valence and self-esteem, while moderation analyses revealed that perceived authenticity buffered against the negative effects of unfavorable feedback. All five preregistered hypotheses were supported.

**Discussion:**

Findings highlight adolescence as a developmental stage of heightened vulnerability to digital evaluation, reflecting neurocognitive imbalance between socio-affective reactivity and regulatory control. Social comparison emerged as a mechanism that amplifies feedback effects, whereas authenticity functioned as a protective factor across all ages. These results refine theoretical models of digital self-esteem regulation and suggest targeted interventions for adolescents, including digital literacy curricula, resilience-building, and platform design modifications to mitigate comparison pressures.

## 1 Introduction

### 1.1 Background and rationale

The widespread use of social network sites has transformed how individuals engage in self-referential processing and identity formation (Collins and Winer, 2024). These digital platforms offer new avenues for self-representation and incorporate feedback cues, such as likes, shares, and comments that provide real-time indicators of social evaluation (Butkowski, 2024). Such feedback loops act as socio-digital mirrors that can mold, sustain, or undermine self-esteem (Hadi et al., 2024). Although a growing body of psychological research has examined the influence of social media on well-being and affect regulation, the age-specific mechanisms of self-esteem regulation in digital contexts remain under-theorized and insufficiently studied from a developmental psychology perspective (Reinecke et al., 2022; Nanjunda, 2010).

Adolescence is characterized by heightened neurobiological plasticity and increased sensitivity to social feedback, making it a critical developmental period for internalizing external evaluations (Cheng et al., 2024). Social media may amplify tendencies toward social comparison and peer conformity by providing constant, quantifiable measures of peer approval (Choukas-Bradley et al., 2022). In contrast, adulthood is generally associated with greater cognitive maturity and self-concept crystallization, which confer enhanced resistance to external validation cues (de Moor et al., 2023). These divergent developmental trajectories suggest that adolescents and adults may differ substantially in emotional reactivity and cognitive appraisal when engaging with self-related digital content. However, few empirical studies have directly compared the effects of social media feedback loops on self-esteem across these age groups (Sonuga-Barke et al., 2024; Resch and Parzer, 2021).

### 1.2 Theoretical foundations

This investigation is grounded in the dual-systems model of socio-emotional development and symbolic interactionist theories of self-concept formation (Casey et al., 2008; Mead, 1934; Goffman, 1959). The dual-systems framework posits that during adolescence, affective reward-processing systems mature earlier than cognitive control systems, creating an asynchrony that heightens sensitivity to the valence of peer feedback, whether positive or negative, particularly in socially evaluative contexts (Jin and Jiang, 2025). From this perspective, feedback valence sensitivity is not merely a byproduct of general self-concept formation but an expected outcome of developmental neurocognitive dynamics (Crone et al., 2022).

The symbolic interactionist perspective complements this view by emphasizing the self as a socially constructed product of ongoing interactions and interpretations of others’ responses (Sundermeier, 2024). In contemporary contexts, these interactions are increasingly mediated through algorithmic platforms that selectively amplify certain types of content and feedback, shaping perceived social norms and influencing which cues are perceived as positive, neutral, or negative (Jin and Jiang, 2025). This selective amplification may intensify social comparison and heighten emotional responses to feedback, particularly when filtered self-representations dominate the online environment.

Empirical evidence suggests that adolescents’ self-esteem is less stable and more susceptible to fluctuations in social acceptance compared to that of adults (Reitz, 2022; Yang et al., 2024). These developmental distinctions likely interact with platform affordances that enhance social comparison and normative influence, especially in feedback-rich environments (West et al., 2025). Linking these theoretical perspectives directly to the study’s hypotheses, the dual-systems model supports the prediction of greater developmental sensitivity to the valence of feedback. At the same time, symbolic interactionism underpins the proposed mediating and moderating roles of social comparison orientation and perceived authenticity (Wahba et al., 2025). Given these theoretical expectations, comparative and developmental research designs are therefore crucial for disentangling age-specific pathways in self-esteem regulation. Yet, few existing studies integrate these perspectives within a single design that simultaneously considers developmental stage, feedback valence, and the cognitive-affective mechanisms of comparison and authenticity. This omission leaves a critical gap in understanding how digital feedback processes may differentially shape self-esteem across the lifespan (Reitz, 2022).

### 1.3 Knowledge gap and significance

Building on this theoretical foundation, we next highlight the empirical gaps that motivate the present study. Although social media use is linked to self-esteem and well-being, few studies have examined how developmental trajectories and platform-specific affordances jointly shape this relationship (Andreassen et al., 2017; Hatchel et al., 2018). Most overlook the bidirectional nature of engagement, in which individuals both receive feedback and actively manage self-presentation in anticipation of audience responses (e.g., deleting posts with low likes, posting content expected to attract approval) (Bareket-Bojmel et al., 2016; Shulman, 2022). Failing to consider this dynamic limits the development of age-appropriate interventions and theoretical models of self-concept in digital contexts (Liu et al., 2025). This study addresses the gap using a multi-method design that combines validated self-report measures and controlled experimental exposure, capturing participants’ responses to feedback within a standardized digital environment.

### 1.4 Research objectives

Despite growing recognition that the developmental stage shapes the psychological impact of social media, existing studies rarely examine adolescents and adults side by side within the same controlled design. Moreover, the mechanisms of social comparison and authenticity have not been systematically integrated into developmental models of online self-esteem regulation. Addressing these gaps provides a clearer lens on how feedback loops operate across the lifespan and why adolescence may represent a period of particular vulnerability. The present study addresses the following central research question:

*How do social media feedback loops differentially affect self-esteem in adolescents compared to adults?*

In this study, we examine the magnitude and directionality of self-esteem responses after exposure to positive, neutral, and negative social media feedback. We also compare feedback sensitivity across adolescents (aged 13–18) and adults (aged 25–40), with these age ranges grounded in developmental psychology literature that identifies adolescence as a period of heightened neurobiological plasticity, socio-emotional reactivity, and identity formation (Casey et al., 2008; Steinberg, 2010), and adulthood as a stage of greater emotional regulation, self-concept stability, and reduced peer conformity (de Moor et al., 2023). Beyond developmental comparisons, we investigate the mediating role of social comparison, i.e., the tendency to evaluate oneself relative to others, which can intensify the emotional consequences of both positive and negative feedback (Festinger, 1954; Vogel et al., 2014). Finally, we assess the moderating role of perceived authenticity, defined as the degree to which individuals’ online self-presentation aligns with their offline identity, as this may buffer against negative self-esteem effects, especially among adults with more coherent self-narratives (Higgins, 1987; Michikyan et al., 2014).

This work employs a multi-method approach combining (a) psychometric self-report data and (b) controlled experimental exposure to manipulated feedback. These complementary methods facilitate a more comprehensive understanding of how developmental stage, cognitive-affective mechanisms, and digital platform dynamics interact to shape self-esteem regulation.

### 1.5 Hypotheses development

#### 1.5.1 Developmental sensitivity to digital feedback across age groups

Adolescence is a sensitive period of socio-affective neurodevelopment, marked by heightened responsiveness to peer evaluation and social feedback (Cheng et al., 2024). Neuroimaging studies have shown that subcortical limbic structures, such as the ventral striatum and amygdala, which play a central role in emotional reactivity and reward processing, undergo rapid maturation during adolescence (Colic et al., 2025; Kumar and Jha, 2024). In contrast, the prefrontal cortex (PFC), which supports cognitive control and emotion regulation, matures more slowly, often not reaching full development until the mid-twenties (Casey et al., 2008; Galván, 2022; Kumar and Jha, 2024). This developmental asynchrony, described by the “imbalance model” (Lichenstein et al., 2022), results in a neurocognitive state in which emotionally charged social cues can exert a disproportionate influence over regulatory control.

Sahi et al. (2023) highlight that adolescents are not only more attuned to peer feedback but also more motivated to seek it as a measure of social standing. In digital contexts, these tendencies are amplified by the constant, quantifiable feedback provided through likes, comments, and reactions (Krasniak et al., 2021). Consequently, adolescents are more likely than adults to experience pronounced boosts in self-esteem following positive feedback and sharper declines following negative feedback. Adults, with more mature regulatory systems and crystallized self-concepts, tend to display greater stability (Mazereel et al., 2024).

*H1 – Developmental Sensitivity Hypothesis:* Adolescents exhibit significantly between-condition differences in post-exposure state self-esteem compared to adults.

#### 1.5.2 Valence sensitivity to social media feedback

Building on the developmental differences outlined in H1, we next consider the direction of feedback effects. From a neuroscience of emotion perspective, the valence of social feedback, positive or negative, has an immediate and measurable impact on self-esteem. Positive feedback, such as likes or affirming comments, activates reward-related brain regions, including the ventral striatum and medial prefrontal cortex, which are associated with reinforcement learning, self-relevant processing, and hedonic evaluation (Dores et al., 2025; Lahiri, 2023). In contrast, negative feedback increases activation in the anterior cingulate cortex (ACC) and insula, areas linked to social pain and rejection (Hill et al., 2022; Eslinger et al., 2021).

Although these mechanisms operate across the lifespan, developmental psychology suggests their salience is particularly high during adolescence due to ongoing identity formation and heightened socio-affective motivation. Adults also experience directional changes, positive feedback raising, and negative feedback lowering self-worth, but often with moderated intensity. Audience engagement and emotional contagion processes can further intensify these effects for both groups (Caspi and Etgar, 2023).

*H2—Valence Main Effect Hypothesis:* Positive feedback leads to significantly higher state self-esteem than neutral or negative feedback across both adolescents and adults.

#### 1.5.3 Age-by-valence interaction in self-esteem modulation

If H1 predicts greater overall variability among adolescents and H2 predicts directional changes across all ages, H3 examines whether the size of these directional changes differs by age. Feedback valence shapes self-perceptions for everyone, but its magnitude appears amplified in adolescence (Pfeifer et al., 2009). Adolescents show more reward-seeking behavior and stronger avoidance of social threats than adults (Fernández-Teruel, 2021). Functional MRI evidence suggests that adolescents exhibit greater ventral striatal activation in response to positive cues and heightened insula responsivity to negative cues (Beard et al., 2022; Pollak et al., 2023).

Experimental studies confirm this developmental divergence. For instance, Nesi and Prinstein (2015) found that adolescents’ emotional responses to manipulated online feedback were significantly stronger than those of emerging adults. This pattern suggests that positive affirmation produces a larger self-esteem gain for adolescents, while negative feedback results in a steeper drop, an age-by-valence interaction consistent with developmental sensitivity and valence processing.

*H3—Development × Valence Interaction Hypothesis:* The effect of feedback valence on state self-esteem is significantly stronger among adolescents than adults.

#### 1.5.4 Social comparison as a mediating mechanism

H1–H3 establishes that adolescents are more sensitive to feedback and that valence effects differ by age. We now turn to why these effects occur. Drawing on Festinger (1954) social comparison theory, people evaluate themselves in relation to others, and social media amplifies this process through constant, public metrics of popularity and validation. Upward social comparison, which involves comparing oneself to idealized peers, is especially common among adolescents and is often associated with lower self-appraisals (McComb et al., 2023; Sun et al., 2023). Adults also engage in social comparison but generally less frequently, with lower emotional intensity, and in a more contextually moderated way. Recent research supports a mediating role for social comparison orientation in the feedback–self-esteem link. Lee (2022) showed that exposure to idealized profiles reduced self-esteem, an effect mediated by comparison orientation, while Tor and Garcia (2023) found that high comparison orientation magnified reactions to inconsistent feedback. These findings suggest that individuals with stronger comparison tendencies may be more vulnerable to the emotional impact of both positive and negative feedback.

*H4—Mediation Hypothesis:* Social comparison orientation mediates the association between feedback valence and post-exposure state self-esteem, such that stronger comparison tendencies are linked to larger between-condition differences in self-perceptions.

#### 1.5.5 Perceived authenticity as a buffering moderator

While H4 identifies a vulnerability pathway, H5 considers a potential protective factor. Instances of digital self-presentation vary in authenticity, and individuals who perceive their online persona as highly aligned with their offline self tend to show lower emotional susceptibility to feedback (Bharathi, 2023). According to self-discrepancy theory (Higgins, 1987), alignment between the actual and ought self promotes psychological equilibrium, whereas misalignment increases vulnerability to feedback-driven distress.

Empirical evidence supports authenticity as a buffer against deception. Individuals presenting exaggerated or inauthentic personas are more prone to distress when engagement is low (Luoma-Aho et al., 2021), whereas those who report authentic self-presentation exhibit reduced variability in emotional reactions to online interactions (Michikyan et al., 2014). Adults are typically more strategic and consistent in self-presentation, potentially making authenticity especially protective for them.

*H5 – Moderation Hypothesis:* The perceived authenticity of digital self-presentation moderates the relationship between feedback valence and self-esteem, buffering against the negative effects of critical feedback across participants, regardless of age group.

**Figure 1:**
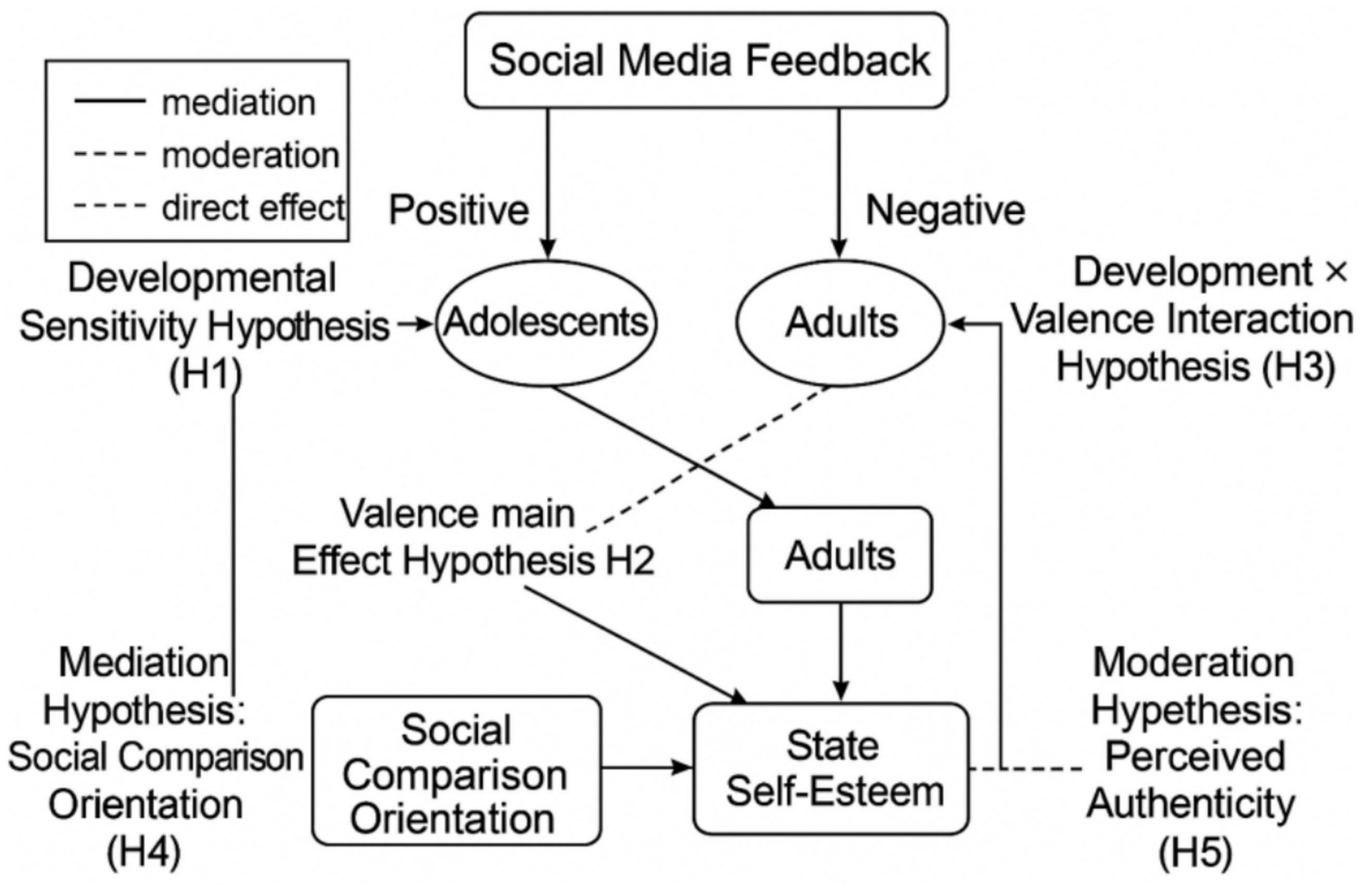
Developmental and cognitive mechanisms linking social media feedback to state self-esteem.

Figure 1 illustrates the hypothesized relationships between social media feedback (positive or negative), age groups (adolescents and adults), and psychological outcomes, with five key hypotheses (H1–H5). H1 suggests adolescents are more sensitive to feedback (developmental sensitivity), while H2 posits a general effect of feedback valence across groups. H3 proposes that the impact of feedback depends on the interaction between developmental stage and valence. H4 tests whether social comparison orientation mediates the association between feedback valence and post-exposure state self-esteem, and H5 examines whether perceived authenticity moderates the link between state self-esteem and outcomes.

## 2 Methods

### 2.1 Study design

The present study employed a cross-sectional, quasi-experimental design to examine developmental differences in the buffering of self-esteem against social media feedback among Chinese adolescents and adults. To balance ecological validity with experimental control, participants were exposed to a simulated social media interface modeled on popular platforms such as WeChat Moments and Xiaohongshu. Within this controlled environment, the valence of feedback (positive, neutral, or negative) was systematically manipulated to assess its impact on self-perception. Order effects and potential participant expectation bias were minimized by randomizing the sequence of feedback presentation and withholding specific study aims until debriefing.

### 2.2 Participants

A sample of 240 respondents was recruited from secondary schools and universities in Beijing, Hangzhou, and Chengdu using stratified purposive sampling, with strata defined by age group. The adolescent sample comprised 120 participants (age range = 13–18 years, M = 15.6, SD = 1.4; 58 females, 62 males) attending junior or senior high school. The adult sample consisted of 120 participants (age range = 25–40 years, M = 32.1, SD = 4.2; 65 females, 55 males), all of whom had at least an undergraduate degree and were either full-time employed or enrolled as postgraduate students.

Inclusion criteria were: (a) active use of at least one social media platform for ≥30 min per day; (b) no self-reported psychiatric diagnosis or ongoing psychological treatment; and (c) no prior participation in studies involving digital self-concept assessment. Written informed consent was obtained from all adult participants and their legal guardians for the adolescent participants. While the stratified design supported group comparability, the sample’s urban location and the high educational attainment of the adult group may limit representativeness, potentially introducing sample bias toward more socioeconomically advantaged and digitally literate populations.

### 2.3 Procedure

Participants were randomly assigned to one of three feedback conditions (positive, neutral, or negative) using a computer-generated randomization schedule. Each participant interacted individually with a gender-neutral, standardized mock profile designed to closely resemble a real user profile on Chinese social media platforms. The profile included a neutral photo, a short biographical blurb, and three posts with manipulated comments and reactions corresponding to the assigned feedback condition. In the negative condition, responses were critical, sarcastic, or disengaged; in the positive condition, comments were affirming and accompanied by high engagement metrics (e.g., “likes,” emojis).

To maintain experimental control, interaction types were limited to likes and comments, intentionally excluding more complex social media features such as shares, private messages, or algorithmic feed changes. This restriction enhanced internal validity but may reduce ecological realism, as actual online environments involve more dynamic and multi-modal interactions. Participants were instructed to imagine that the mock profile represented their own and that the feedback reflected responses from their real social network. They had 5 min to familiarize themselves with the profile before completing a battery of psychometric questionnaires assessing momentary self-esteem, cognitive appraisals, and emotional responses. The entire experimental procedure lasted approximately 25 min.

### 2.4 Measures

State Self-Esteem was assessed using the Mandarin Chinese version of the State Self-Esteem Scale (SSES) (Heatherton and Polivy, 1991); Chinese adaptation by Fung et al. (2006), which comprises three subscales: performance self-esteem, social self-esteem, and appearance self-esteem. Items were rated on a 5-point Likert scale (1 = “not at all,” 5 = “extremely”), with higher scores indicating greater momentary self-esteem. Two items were reverse-coded to reduce acquiescence bias. Internal consistency was high in both adolescents (*α* = 0.89) and adults (α = 0.91), consistent with previous cross-cultural validations. Social Comparison Orientation was measured using the Iowa–Netherlands Comparison Orientation Measure (INCOM; Gibbons and Buunk, 1999), which has demonstrated robust psychometric properties among East Asian samples. The scale assesses tendencies toward comparison in ability and opinion domains, using a 5-point Likert scale (1 = strongly disagree, 5 = strongly agree). Three items were reverse-coded. Reliability in the present sample was α = 0.84.

Perceived authenticity of digital self-presentation was evaluated using a 5-item scale adapted from Marwick (2013), revised for cultural appropriateness and familiarity with Chinese social media norms, following the adaptation procedure outlined by Cui et al. (2024). Items assessed the perceived congruence between participants’ online and offline selves, the genuineness of their self-presentation, and its perceived social appropriateness, rated on a 7-point Likert scale (1 = strongly disagree, 7 = strongly agree). Internal consistency was α = 0.81. The digital usage profile included platform preferences (WeChat, Douyin, Weibo, Xiaohongshu), average daily social media use (in minutes), and feedback monitoring frequency. Feedback monitoring was operationalized as the self-reported number of times participants checked for “likes” or comments in a typical day, measured via a single-item frequency question (1 = never, 5 = more than 10 times per day).

### 2.5 Manipulation checks and baseline comparability

Before testing hypotheses, we first examined whether random assignment produced comparable groups across the three feedback conditions (positive, neutral, negative). One-way ANOVAs were conducted for continuous variables (age, daily social media use, and feedback monitoring frequency), and chi-square tests were used to examine the distribution of genders. No significant differences were found on any demographic or usage variable (*p* values > 0.10), indicating balanced groups prior to exposure. Next, to verify the effectiveness of the feedback manipulation, participants rated the perceived valence of the feedback (“How positive or negative was the feedback you saw?”; 1 = very negative, 4 = neutral, 7 = very positive) and the perceived credibility/intensity of the feedback (“How believable and emotionally impactful was the feedback you saw?”; 1 = not at all, 7 = extremely). One-way ANOVAs confirmed significant differences in perceived valence across conditions (*p* < 0.001), with positive feedback rated as more positive, neutral feedback as intermediate, and negative feedback as more negative. Credibility/intensity ratings were also significantly higher in the positive and negative conditions than in the neutral condition (*p* < 0.001). These results confirm that the randomization procedure produced demographically and behaviorally equivalent groups at baseline and that the feedback manipulation was both directionally effective and perceived as credible and impactful (Table 1).

**Table 1:** Baseline characteristics and manipulation check results by feedback condition.

Variable	Positive (*n* = 80)	Neutral (*n* = 80)	Negative (*n* = 80)	Test statistic	*p* value
Age, M (SD)	23.9 (7.8)	24.1 (7.5)	24.3 (7.7)	F(2, 237) = 0.05	0.95
Gender (% female)	50.0	52.5	51.3	χ^2^(2) = 0.07	0.96
Daily social media use (min/day)	128.5 (39.2)	131.0 (40.8)	129.4 (38.7)	F(2, 237) = 0.09	0.91
Feedback monitoring frequency	3.1 (1.1)	3.0 (1.0)	3.1 (1.2)	F(2, 237) = 0.18	0.84
Perceived valence M (SD)	6.21 (0.68)	4.02 (0.59)	1.89 (0.72)	F(2, 237) = 802.35	<0.001
Credibility/intensity M (SD)	5.87 (0.81)	4.11 (0.77)	5.64 (0.84)	F(2, 237) = 92.14	<0.001

### 2.6 Data analysis

All statistical analyses were performed using SPSS version 28 and Mplus version 8.0. Data analysis: Descriptive statistics, preliminary normality, and homoscedasticity assumption checks were conducted before testing the hypothesis. A two-way ANOVA was used to analyze the influence of age group (adolescents vs. adults) and feedback valence (positive vs. neutral vs. negative) on post-exposure self-esteem scores in the post-exposure phase. Pairwise Tukey’s HSD post-hoc comparisons were performed to analyze the main effects. To investigate the mediating psychological mechanisms, mediation analyses using the bootstrap method (5,000 resamples) were conducted with bias-corrected confidence intervals. Social comparison orientation and perceived authenticity were examined as mediators of the relationship between feedback conditions and self-esteem effects. Gender and average daily social media consumption were entered as covariates in all models, as these have been reported to affect online self-concept dynamics. Because the moderation analysis did not include a three-way Age × Valence × Authenticity interaction, moderation effects are interpreted as applying across participants as a whole rather than separately by age group.

## 3 Results

### 3.1 Reliability and validity analysis

Cronbach’s alpha coefficient, the Kaiser-Meyer-Olkin (KMO) measure, and Bartlett’s sphericity test were calculated to assess the internal consistency and construct validity of the instruments. As shown in Table 2, all measures demonstrated good reliability, with Cronbach’s alpha coefficients ranging from 0.81 to 0.91, all of which exceeded the 0.70 criterion for psychological measurements. KMO values were also high (0.79–0.89) and indicated adequate sampling for factor analysis. Bartlett’s test was significant (*p* < 0.001) for all instruments, indicating that the data were appropriate for multivariate analysis. These results demonstrate the statistical reliability and structural validity of the psychometric instruments.

**Table 2:** Reliability and validity measures.

Scale	Cronbach alpha	KMO measure	Bartlett’s test *p*-value
State self-esteem (SSES)	0.91	0.89	< 0.001
Social comparison orientation (INCOM)	0.84	0.83	< 0.001
Perceived authenticity scale	0.81	0.79	< 0.001

### 3.2 Assumption testing: normality

We used the Shapiro–Wilk test to assess the normality assumption for the key continuous variables. As shown in Table 3, all test *p*-values were greater than 0.05 (range = 0.076–0.102), indicating no significant deviations from normality. W statistics were close to 1.0 (e.g., W = 0.98 for State Self-Esteem), further supporting the assumption. Levene’s tests for equality of variances indicated that the homogeneity of variance assumption was met for all ANOVA models (*p* > 0.05), and Brown–Forsythe tests yielded the same conclusion. Given that neither normality nor homogeneity assumptions were violated, no Welch or other robust corrections were required. These findings supported the applicability of parametric statistics, such as ANOVA and regression, in the subsequent hypothesis testing.

**Table 3:** Normality and homogeneity of variance checks.

Variable	W statistic	*p*-value (normality)	Levene’s *F*	*p*-value (homogeneity)
State self-esteem	0.98	0.08	1.21	0.30
Social comparison orientation	0.97	0.10	0.94	0.39
Perceived authenticity	0.97	0.07	0.88	0.42

### 3.3 Descriptive statistics by age group and feedback condition

Table 4 presents descriptive statistics (means ± SDs) for post-exposure state self-esteem across the Age × Feedback Valence cells. Adolescents generally showed larger score shifts between positive and negative conditions compared to adults.

**Table 4:** Post-exposure state self-esteem (M ± SD) by age group and feedback valence.

Age group	Positive feedback (*n* ≈ 40)	Neutral feedback (n ≈ 40)	Negative feedback (*n* ≈ 40)
Adolescents	4.25 ± 0.52	3.87 ± 0.48	3.12 ± 0.56
Adults	4.05 ± 0.49	3.92 ± 0.50	3.45 ± 0.54

Higher scores indicate greater momentary state self-esteem. Ns are approximate based on equal random assignment; exact Ns should be reported if slightly unequal.

### 3.4 Hypothesis testing

Table 5 shows that age had a significant effect on post-feedback state self-esteem, *F*(1, 238) = 28.27, *p* < 0.001, with a large effect size (η^2^ = 0.11). Adolescents reported greater sensitivity in self-esteem than adults, supporting the developmental sensitivity hypothesis (H1).

**Table 5:** ANOVA results for hypothesis 1 (developmental sensitivity).

Source	SS	*df*	MS	*F*	*p*	η^2^
Between groups (age)	112.30	1	112.30	28.27	< 0.001	0.11
Within groups	945.60	238	3.97	-	-	-
Total	1057.90	239	-	-	-	-

Table 6 reveals a significant main effect of feedback valence on state self-esteem, *F*(2, 237) = 10.85, p < 0.001, with a medium effect size (η^2^ = 0.08). Participants who received positive feedback reported higher self-esteem than those in neutral or negative feedback conditions, confirming the valence main effect hypothesis (H2).

**Table 6:** ANOVA results for hypothesis 2 (valence main effect).

Source	SS	*df*	MS	*F*	*p*	η^2^
Between groups (valence)	88.70	2	44.35	10.85	< 0.001	0.08
Within groups	969.20	237	4.09	-	-	-
Total	1057.90	239	-	-	-	-

Table 7 shows a significant Age × Valence interaction, *F*(2, 234) = 6.65, *p* = 0.002, with a small-to-medium effect size (η^2^ = 0.05). This indicates that the impact of feedback valence on state self-esteem varied by age, with adolescents showing larger increases after positive feedback and greater decreases after negative feedback compared to adults, supporting the interaction hypothesis (H3).

**Table 7:** Two-way ANOVA results for hypothesis 3 (age × valence interaction).

Source	SS	*df*	MS	*F*	*P*	η^2^
Age	112.30	1	112.30	32.36	< 0.001	0.12
Valence	88.70	2	44.35	12.78	< 0.001	0.10
Age × valence	46.20	2	23.10	6.65	0.002	0.05
Error	810.70	234	3.47	-	-	-
Total	1057.90	239	-	-	-	-

The mediation analysis (Table 8) indicated that, relative to the neutral feedback condition, both positive and negative feedback significantly predicted higher social comparison orientation (Positive vs. Neutral: *B* = 0.34, *p* < 0.001, *β* = 0.29; Negative vs. Neutral: *B* = 0.30, *p* = 0.001, β = 0.26). In turn, higher social comparison orientation significantly predicted lower state self-esteem (*B* = −0.45, *p* < 0.001, *β* = −0.35). Bootstrapped indirect effects were significant for both contrasts (Positive vs. Neutral: *B* = −0.15, 95% CI [−0.25, −0.06], *p* = 0.003; Negative vs. Neutral: *B* = −0.14, 95% CI [−0.24, −0.05], *p* = 0.004), confirming partial mediation of the valence–self-esteem relationship through social comparison. Direct effects of feedback valence on self-esteem remained significant but were smaller in magnitude (Positive vs. Neutral: *B* = 0.22, *p* = 0.015, *β* = 0.20; Negative vs. Neutral: *B* = 0.20, *p* = 0.019, *β* = 0.18), supporting the mediation hypothesis (H4) while indicating that other unmeasured factors may also contribute to the effect.

**Table 8:** Mediation analysis.

Path	B	SE	*p*-value	95% CI	Effect size (β)
Positive vs. neutral → social comparison	0.34	0.09	<0.001	[0.16, 0.52]	0.29
Negative vs. neutral → social comparison	0.30	0.09	0.001	[0.12, 0.48]	0.26
Social comparison → self-esteem	−0.45	0.07	<0.001	[−0.59, −0.31]	−0.35
Indirect effect (positive vs. neutral)	−0.15	0.05	0.003	[−0.25, −0.06]	-
Indirect effect (negative vs. neutral)	−0.14	0.05	0.004	[−0.24, −0.05]	-
Direct effect (positive vs. neutral)	0.22	0.09	0.015	[0.04, 0.40]	0.20
Direct effect (negative vs. neutral)	0.20	0.09	0.019	[0.03, 0.37]	0.18

Valence was dummy-coded with Neutral as the reference group (two contrasts: positive vs. neutral, negative vs. neutral). Indirect effects were estimated using 5,000 bootstrapped samples with bias-corrected 95% confidence intervals. All *p*-values are two-tailed. All coefficients are adjusted for gender and average daily social media use.

The moderation analysis (Table 9) indicated that both feedback valence (*B* = 0.27, *p* = 0.003) and perceived authenticity (*B* = 0.35, *p* < 0.001) significantly predicted state self-esteem. The significant Valence × Authenticity interaction (*B* = 0.19, *p* = 0.007) suggests that higher authenticity buffered the negative impact of less favorable feedback on state self-esteem across the full sample.

**Table 9:** Moderation analysis (perceived authenticity).

Model term	B	SE	T	*p*
Feedback valence	0.27	0.09	3.00	0.003
Perceived authenticity	0.35	0.08	4.38	<0.001
Valence × authenticity interaction	0.19	0.07	2.71	0.007

The results of all five hypotheses tested are presented in Table 10, and all received significant support. Results provided further evidence that the level of variance in self-esteem is higher among youth compared to adult age groups regarding social media feedback (H1) and that feedback valence significantly affects self-esteem, with positive feedback exerting a more substantial effect, regardless of age (H2). A meaningful interaction effect revealed that adolescents are more sensitive to feedback valence than adults (H3). Moreover, social comparison orientation was a significant mediator of the effect of feedback valence on self-esteem (H4), and perceived authenticity moderated the relationship, mitigating the negative consequences of critical feedback across the sample (H5). These findings support the developmental and cognitive-affective processes posited by the conceptual model in the current study.

**Table 10:** Summary of hypothesis testing results.

Hypothesis	Supported?	Statistical test	Key statistic
H1–Developmental sensitivity	Yes	One-way ANOVA	*F* (1,238) = 28.27, *p* < 0.001
H2–Valence main effect	Yes	One-way ANOVA	*F* (2,237) = 10.85, *p* < 0.001
H3–Age Ã, valence interaction	Yes	Two-way ANOVA	*F* (2,234) = 6.65, *p* = 0.002
H4–Mediation (social comparison)	Yes	Mediation analysis (bootstrapped)	Indirect Effect B = -0.14, 95% CI [−0.24, −0.05], *p* = 0.004
H5 –Moderation (authenticity)	Yes	Moderated regression	Interaction B = 0.19, *p* = 0.007

## 4 Discussion

The present study set out to address a central question identified in the Introduction: ‘*How do social media feedback loops differentially affect self-esteem in adolescents compared to adults?*’ By situating this question within developmental and cognitive-affective frameworks, and by responding to gaps in prior research that rarely examined adolescents and adults side by side, the study provides new evidence on how feedback valence, social comparison, and authenticity jointly shape post-exposure self-esteem. The results of this study support the proposed role of age, feedback valence, and mediating/moderating psychological characteristics in moderating the effects of digital experiences on self-esteem.

### 4.1 Developmental divergences in feedback sensitivity

Consistent with the Developmental Sensitivity Hypothesis (H1), adolescents demonstrated significantly larger between-condition differences in post-exposure state self-esteem than adults. This supports neurodevelopmental models that describe adolescence as a period of heightened socio-affective reactivity, accompanied by incomplete regulatory control (Tomova et al., 2021; Steinberg, 2010). Our findings extend these models by showing that heightened sensitivity is not limited to face-to-face peer interactions, but is equally, if not more pronounced, in digital contexts where feedback is quantifiable and persistent. In Chinese adolescents, this reactivity may be amplified by cultural factors such as collectivist values, strong norms around “face” preservation, and academic pressures that heighten the salience of peer approval or disapproval in shaping self-worth. By contrast, adults’ relatively stable self-concept and more mature regulatory systems (Mouatsou and Koutra, 2023; Martín Quintana et al., 2023) appear to buffer against rapid fluctuations, reflecting a developmental progression toward resilience in socio-evaluative contexts.

### 4.2 The direct and interactive influence of feedback valence

The Valence Main Effect Hypothesis (H2) was supported: across both age groups, positive feedback increased, and negative feedback decreased, resulting in increased state self-esteem. This aligns with theories positioning social media as a “digital mirror” (Merino et al., 2024; Khan et al., 2023), extending prior findings by quantifying the magnitude of these effects in a controlled setting. However, the Age × Valence Interaction Hypothesis (H3) revealed that adolescents’ self-esteem gains from positive feedback and losses from negative feedback were substantially greater than those observed in adults. This developmental amplification is consistent with evidence of heightened reward sensitivity and threat reactivity during adolescence (Nesi and Prinstein, 2015; Sherman et al., 2016), and it refines interactionist perspectives by demonstrating that the same valenced stimulus can produce asymmetrical psychological impacts depending on the developmental stage. In China’s high-achievement educational climate, such patterns may be magnified, as public digital approval or criticism can carry both social and academic implications.

### 4.3 Psychological mechanisms: social comparison and authenticity

Two individual difference variables helped explain these patterns. First, as predicted by the Mediation Hypothesis (H4), social comparison orientation significantly mediated the relationship between feedback and self-esteem. Participants higher in comparison orientation, especially adolescents, were more likely to engage in upward comparisons with idealized peers, which amplifies the adverse effects of critical feedback and enhances the benefits of positive feedback (Festinger, 1954; Vogel et al., 2014; Krogh, 2023; Tiggemann and Anderberg, 2020). This extends social comparison theory by situating it within algorithmically curated environments, where selective self-presentations are pervasive and exposure is continuous.

Second, the Moderation Hypothesis (H5) was supported: perceived authenticity of digital self-presentation buffered against the harmful effects of negative feedback across the sample. This extends self-discrepancy theory (Higgins, 1987) by showing that greater alignment between online and offline selves can reduce emotional volatility in digital feedback contexts. In collectivist cultures, where harmony and self-presentation norms are salient, authenticity may act as a resilience factor, helping individuals interpret criticism as less identity-threatening. These findings suggest that developmental sensitivity (H1) shapes the context in which valence effects (H2) and age–valence interactions (H3) emerge, with mediation by social comparison (H4) and moderation by authenticity (H5) further refining the patterns. Intervention strategies could target adolescent digital resilience by promoting critical reflection on feedback, strengthening emotion regulation skills, and encouraging internal rather than external attribution styles for online evaluation. In the Chinese context, incorporating these approaches into educational curricula could help mitigate socio-digital reactivity and promote healthier online self-presentation habits.

### 4.4 Theoretical and practical implications

Theoretically, this study contributes to the expanding literature on developmental cyberpsychology and the integration of neurodevelopmental, cognitive-affective, and social-contextual perspectives on digital self-regulation. It adds nuance to how age-specific vulnerabilities intersect with the affordances of the platform and individual psychological characteristics to explain diverging self-esteem profiles.

From a practical point of view, the results imply that we should not adopt a single-model strategy for digital literacy programs. Interventions should address comparison-based engagement and consider promoting digital resilience and authentic self-presentation in adolescents. For adults, the focus may be on promoting healthy identity coherence and discouraging identification with online metrics.

### 4.5 Limitations and future research

The present study has several limitations. Although the simulated social media environment enhanced experimental control, it lacked the complexity and contextual richness of real-world platforms, potentially reducing ecological validity and introducing demand characteristics if participants inferred the study’s purpose. The standardized profile, while neutral, may have limited realism compared to authentic user interactions. In addition, although participants reported their frequency of feedback monitoring, the study did not include directly logged behavioral indicators during the task. As such, the design should be regarded as multi-method rather than fully triangulated.

Moreover, the reliance on post-exposure self-reported state self-esteem without a pre-test/post-test design raises the possibility of trait–state confounds, as baseline differences could have influenced outcomes despite random assignment. Accordingly, the present design permits inference about between-group and between-condition differences at the post-exposure stage, but does not allow conclusions about within-person fluctuations over time.

Future research should incorporate baseline and repeated measures, longitudinal designs, and neurophysiological indicators to capture cumulative and real-time processes. Finally, the Chinese cultural context, characterized by collectivism, face-saving norms, and high-context communication, may heighten sensitivity to evaluative feedback, underscoring the need for cross-cultural replications to assess generalizability.

## 5 Policy and practical implications

This study has important implications for policymakers, educators, mental health practitioners, and platform developers concerned with digital well-being. Adolescents’ vulnerability to self-esteem instability in response to online feedback suggests that age-specific psychoeducation may be a valuable digital mental health strategy in educational settings. Curriculum writers could integrate media literacy modules to raise awareness of social comparison biases and promote genuine self-presentation as protective factors against these biases. At the policy level, youth internet governance frameworks in China and other contexts might consider interventions that regulate the visibility of feedback (e.g., hiding public “like” counts or enabling private feedback modes) to reduce dependence on social validation. However, such measures face significant implementation challenges, including platform compliance, varying jurisdictional regulations, and the potential for unintended consequences, as private feedback channels may still perpetuate comparison and exclusion in subtler forms. Parental guidance initiatives should be supported with evidence-based toolkits that facilitate open dialogue about peer comparison and self-esteem in digital contexts. From a technological design perspective, platform engineers and UX designers could prioritize features that foster substantive, narrative-based interactions rather than solely metric-driven engagement, supporting healthier identity development and reducing validation anxiety. While adults may be less reactive, they too could benefit from design elements that encourage authentic self-presentation and reinforce emotional boundaries online.

## 6 Conclusion

The present results contribute to convergent evidence that social media feedback has a differential psychological impact across the lifespan, with adolescents exhibiting heightened emotional sensitivity to validation and criticism compared to adults. Feedback valence significantly affects state self-esteem, and this relationship is mediated by social comparison orientation and moderated by perceived authenticity of self-presentation. By combining developmental psychology, digital behavior science, and social cognitive theory, this study extends our understanding of the interdependence of age, cognition, and identity in socially mediated contexts. These findings have significant implications for digital policy, platform design, education, and mental health, underscoring the need for more personalized, culturally sensitive interventions to promote digital well-being. As the world grows increasingly digital, the intersection between psychological development and technological design will be pivotal for preserving mental health and identity formation in the next generation.

## Data Availability

The original contributions presented in the study are included in the article/supplementary material, further inquiries can be directed to the corresponding author.
